# Automatic amygdala response to facial expression in schizophrenia: initial hyperresponsivity followed by hyporesponsivity

**DOI:** 10.1186/1471-2202-14-140

**Published:** 2013-11-13

**Authors:** Thomas Suslow, Christian Lindner, Udo Dannlowski, Kirsten Walhöfer, Maike Rödiger, Birgit Maisch, Jochen Bauer, Patricia Ohrmann, Rebekka Lencer, Pienie Zwitserlood, Anette Kersting, Walter Heindel, Volker Arolt, Harald Kugel

**Affiliations:** 1 Department of Psychiatry, University of Münster, Albert-Schweitzer-Str. 11, Münster 48149, Germany; 2 Department of Psychosomatic Medicine, University of Leipzig, Semmelweisstr 10, Leipzig 04103, Germany; 3 Department of Psychiatry, University of Marburg, Rudolf-Bultmann-Str. 8, Marburg 35037, Germany; 4 Klinik am Schlossgarten, Am Schlossgarten 10, Dülmen 48249, Germany; 5 Department of Psychology, University of Münster, Fliednerstr. 21, Münster 48149, Germany; 6 Department of Clinical Radiology, University of Münster, Albert-Schweitzer-Campus 1, Münster 48149, Germany

**Keywords:** Schizophrenia, Automatic processing, fMRI, Amygdala, Facial expression, Temporal dynamics

## Abstract

**Background:**

It is well established that the amygdala is crucially involved in the processing of facial emotions. In schizophrenia patients, a number of neuroimaging findings suggest hypoactivation of the amygdala in response to facial emotion, while others indicate normal or enhanced recruitment of this region. Some of this variability may be related to the baseline condition used and the length of the experiment. There is evidence that schizophrenia patients display increased activation of the amygdala to neutral faces and that this is predominantly observed during early parts of the experiment. Recent research examining the automatic processing of facial emotion has also reported amygdala hyperactivation in schizophrenia. In the present study, we focused on the time-course of amygdala activation during the automatic processing of emotional facial expression. We hypothesized that in comparison to healthy subjects, patients would initially show hyperresponsivity of the amygdala to masked emotional and neutral faces. In addition, we expected amygdala deactivation in response to masked facial emotions from the first to the second phase to be more pronounced in patients than in controls.

**Results:**

Amygdala activation in response to angry, happy, neutral, and no facial expression (presented for 33 ms) was measured by functional magnetic resonance imaging in 30 schizophrenia patients and 35 healthy controls. Across all subjects, the bilateral amygdala response to faces (relative to the no facial expression condition) was larger in the initial phase (first half of trials) than in the second phase (second half of trials). During the initial phase, schizophrenia patients exhibited an increased right amygdala response to all faces and an increased left amygdala response to neutral faces compared with controls. During the second phase, controls manifested a higher right amygdala response for all faces and a higher left amygdala response to angry faces than patients.

**Conclusions:**

Schizophrenia patients are characterized by high initial amygdala responsivity to facial expressions at an automatic processing level, which substantially decreases with time. Amygdala deactivation over time might reflect an automatic mechanism by which schizophrenia patients suppress the processing of facial stimuli. This blocking mechanism could help patients avoid overstimulation during social interactions.

## Background

Over the last few decades, many studies have reported abnormalities in the processing of facial emotions in schizophrenia patients. Most studies find that patients show significant deficits in facial affect recognition [1], which may be related to poor social functioning [2]. With respect to the neural underpinnings of these deficits, research has focused on the limbic system and, in particular, on the amygdala [3]. It has been repeatedly shown that the amygdala is crucially involved in the perception of facial emotions [4]. Findings from neuroimaging studies on the amygdala response to facial expression in schizophrenia are heterogeneous and strongly dependent on experimental design. Some authors have found that patients show reduced activation of the amygdala in response to emotional face stimuli, which could be interpreted as an impairment in the processing of affective information [5,6]. On the other hand, others have shown normal activation or even hyperactivation of the amygdala, indicating that patients may suffer from hypersensitivity to emotional faces [7-9].

Thus far, only a few studies have investigated the automatic processing of facial emotion and its neural correlates in schizophrenia. This is surprising because emotional reactions are generally elicited involuntarily and emerge without conscious effort [10]. Two behavioral studies have examined the effect of masked facial emotion on evaluation processes using an affective priming paradigm [11]. Affective priming paradigms can be applied to examine whether facial emotional expression presented outside of conscious awareness colors evaluative judgments. Typically, neutral stimuli preceded by subliminally shown happy faces are evaluated more positively than those preceded by subliminally shown neutral faces. Neutral stimuli preceded by subliminally presented angry faces are evaluated more negatively than those preceded by subliminally presented neutral faces [11]. This phenomenon of an affective influence of emotional facial expression on subsequent judgments is termed the *affective priming effect*. Höschel and Irle [12] were the first to demonstrate that schizophrenia patients exhibit a stronger negative evaluative bias in response to covert negative facial expression (i.e., more negative affective priming) than healthy controls. Extending these findings, Suslow et al. [13] observed that schizophrenia patients suffering from affective negative symptoms (i.e., anhedonia or flat affect) were particularly sensitive to negative facial expressions on an automatic processing level. It was concluded that social withdrawal and symptoms of flat affect in schizophrenia might develop as self-protective mechanisms to reduce aversive stimulation during social interactions.

To our knowledge, only two neuroimaging studies have examined the automatic processing of facial emotion in schizophrenia. According to the results of Das et al. [14], schizophrenia patients show reduced activity in the bilateral amygdala during perception of masked fearful faces (relative to neutral faces) compared to healthy controls. The observed amygdala hypoactivation could be due to the baseline condition used (i.e., neutral faces) because previous research indicates that schizophrenia patients exhibit increased activation of the amygdala to neutral facial expressions [15]. In a subsequent fMRI study [16], schizophrenia patients showed stronger activation of the amygdala in response to sad and happy faces than did healthy controls. However, the effects were quite small, potentially due to the small sample sizes studied (N = 12 for patients and controls).

Because previous findings regarding the automatic amygdala response to facial emotion have been inconsistent, further research on this issue is required. The first aim of the present study was to examine whether schizophrenia patients manifest amygdala hyperactivation to facial expression at an automatic processing level. Automaticity of processing can be assumed when processing occurs rapidly, unintentionally, and with no or minimal attention [17]. In our fMRI experiment, we presented angry, happy, and neutral faces as stimuli only very briefly; these were subsequently masked by neutral faces. We used no facial expression stimuli as the baseline condition because schizophrenia patients have previously demonstrated increased amygdala responses to neutral faces [3,15].

Our second aim was to investigate the time-course of the neural processing of facial emotion. It has been shown that affective priming with masked emotional faces emerges strongly in initial presentations but decreases rapidly with repetition [18]. Moreover, it is well established that the amygdala response habituates (i.e., decreases over time) to repeated presentations of facial expressions [19,20]. Finally, it has been observed that patients with schizophrenia show similar or higher amygdala activation in response to facial emotions during the initial stage of processing than healthy controls but that the activation in patients appears to decrease more strongly with repetition [9,15]. Thus, there are several reasons to investigate the automatic processing of facial emotion as a function of time in both schizophrenia patients and healthy individuals. According to Holt and Phillips [21], there is the possibility that changes in brain function during emotional processing in schizophrenia are dynamic in nature, with an initial hyperresponsivity followed by hyporesponsivity of medial temporal lobe structures, including the amygdala. To investigate the time-course of automatic emotion processing, our fMRI priming experiment was split into two phases of equal length in which identical facial stimuli were displayed.

We hypothesized that compared to controls, patients would initially manifest heightened amygdala activation in response to all facial expressions (versus no facial expression). Second, we expected that both schizophrenia patients and controls would show stronger amygdala activation to angry, happy, and neutral faces during the initial phase than during the second phase of the experiment. Third, we hypothesized that amygdala deactivation from the initial to the second phase would be more pronounced for patients than controls.

## Results

### Behavioral results

Table 1 shows mean evaluative ratings and affective priming scores of patients and controls for all priming conditions during both phases of the experiment. According to the results from the 2*2*3 ANOVA, there was no significant effect of group (F(1,63) = 0.039, p = 0.844) or phase (F(1,63) = 0.001, p = 0.975), but the effect of condition was significant (F(2,126) = 6.158, p = 0.003). The interactions between condition and group (F(2,126) = 1.497, p = 0.228) and between phase and group (F(1,63) = 0.025, p = 0.667) were not significant, but we observed a significant interaction between phase and condition (F(2,126) = 10.095, p < 0.001) and a significant group*phase*condition interaction (F(2,126) = 3.290, p = 0.041).

**Table 1:** Mean evaluative ratings and affective priming scores of patients and controls during the initial phase and second phase of the experiment (patients: N = 30; controls: N = 35); mean ± SE; range of evaluative ratings: -1.5 to +1.5; affective priming score: difference between rating of facial prime condition and rating of no facial expression baseline condition

		**Patients**	**Controls**
**Mean evaluative ratings**			
No facial expression	Initial	0.00 ± 0.27	−0.07 ± 0.28
	Second	0.13 ± 0.33	0.02 ± 0.26
Neutral	Initial	−0.04 ± 0.25	−0.06 ± 0.21
	Second	−0.05 ± 0.27	−0.04 ± 0.25
Angry	Initial	−0.17 ± 0.24	−0.12 ± 0.24
	Second	−0.11 ± 0.30	−0.02 ± 0.27
Happy	Initial	0.01 ± 0.28	−0.09 ± 0.25
	Second	0.14 ± 0.31	0.02 ± 0.27
**Affective priming scores**			
Neutral	Initial	0.04 ± 0.34	0.01 ± 0.25
	Second	−0.08 ± 0.34	−0.06 ± 0.15
Angry	Initial	−0.17 ± 0.32	−0.05 ± 0.20
	Second	−0.01 ± 0.28	−0.04 ± 0.17
Happy	Initial	0.00 ± 0.31	−0.02 ± 0.20
	Second	0.01 ± 0.22	0.00 ± 0.14

Subsequently, 2 (group)*2 (phase) ANOVAs were conducted for all affective priming conditions (i.e., angry, happy, and neutral). The results from the 2*2 ANOVA for angry primes indicated a significant effect of phase (F(1,63) = 4.487, p = 0.038), as mean evaluative ratings were more positive during the second phase. The main effect of group was not significant (F(1,63) = 1.008, p = 0.319) but the interaction between phase and group was close to significance (F(1,63) = 3.495, p = 0.066). According to the results from independent two sample t-tests, negative priming for masked angry faces tended to be stronger in patients than in controls during the initial phase (t = −1.77, df = 63, p = 0.083) but was similar during the second phase (t = 0.50, df = 63, p = 0.622).

According to the results from the 2 (group)*2 (phase) ANOVA for happy primes, there were no significant effects of phase (F(1,63) = 0.106, p = 0.746) or group (F(1,63) = 0.281, p = 0.598) nor was there a significant group*phase interaction (F(1,63) = 0.027, p = 0.871).

The results from the 2 (group)*2 (phase) ANOVA for neutral primes indicated a marginally significant effect of phase (F(1,63) = 3.714, p = 0.058). Priming scores for neutral faces were more negative during the second phase compared to the initial phase. Neither the effect of group (F(1,63) = 0.009, p = 0.925) nor the interaction between phase and group (F(1,63) = 0.176, p = 0.676) were significant.

### fMRI results

#### Full factorial ANOVA model: group*phase*condition

The results from the 2 (group: schizophrenia patients and healthy controls)*2 (phase: first and second half)*3 (condition: angry, happy, and neutral) full factorial ANOVA revealed no effect of group on amygdala activation. The effect of phase on amygdala activation was significant (left peak voxel xyz: -20 -4 -20, cluster size: 128, F(1,378) = 28.11, Z-score = 5.07, p = 0.0000002; right peak voxel xyz: 22 0–20, cluster size: 104, F(1,378) = 12.81, Z-score = 3.36, p =0.0004), indicating that amygdala activation was larger during the initial phase than in the second phase. Condition showed an effect on right amygdala activation (peak voxel xyz: 24–4 -18, cluster size: 45, F(2,378) = 6.81, Z-score = 3.02, p = 0.001). The group*condition and phase*condition interaction effects on amygdala activation were not significant, but the interaction between group and phase was significant (left peak voxel xyz: -26 -2 -12, cluster size: 75, F(1,378) = 12.02, Z-score = 3.24, p = 0.0006; right peak voxel xyz: 26–4 -14, cluster size: 81, F(1,378) = 18.37, Z-score = 4.07, p = 0.00002). Furthermore, the group*phase*condition interaction effect was significant for the left amygdala (peak voxel xyz: -28 -6 -14, cluster size: 58, F(2,378) = 9.75, Z-score = 3.79, p = 0.00008).

### Post-hoc analyses

#### Activation of the left amygdala in response to angry faces as a function of group and phase

Results from a 2 (group)*2 (phase) ANOVA indicate a significant effect of phase on left amygdala activation, with higher amygdala recruitment during the initial phase of the experiment (peak voxel xyz: -20 -6 -18, cluster size: 114, F(1,126) = 28.17, Z-score = 4.90, p = 0.0000005). Group status had no significant main effect on amygdala activation but the interaction between group and experimental phase on left amygdala activation was highly significant (peak voxel xyz: -24 -2 -14, cluster size: 97, F(1,126) = 19.26, Z-score = 4.07, p = 0.00002).

Subsequent post-hoc t-tests showed that controls manifested higher left amygdala activation during the second (but not the first) phase of the experiment than patients (peak voxel xyz: -24 -4 -16, cluster size: 118, t(63) = 4.40, Z-score = 4.09, p = 0.00004). Patients exhibited no stronger activation than controls in response to angry faces.

#### Activation of the left amygdala in response to happy faces as a function of group and phase

A group*phase ANOVA revealed a significant effect of phase on left amygdala activation (peak voxel xyz: -20 -4 -18, cluster size: 111, F(1,126) = 29.49, Z-score = 5.01, p = 0.0000003), indicating that the left amygdala response to happy faces was larger in the initial phase than in the second phase. Group status had no significant effect on amygdala activation, and the interaction between group and phase was also not significant.

#### Activation of the left amygdala in response to neutral faces as a function of group and phase

A group*phase ANOVA again revealed a significant effect of phase on left amygdala activation (peak voxel xyz: -24 2–16, cluster size: 67, F(1,126) = 10.80, Z-score = 3.01, p = 0.001). Group status had no significant effect on left amygdala activation, but the interaction between group and phase was significant (peak voxel xyz: -26 -2 -12, cluster size: 51, F(1,126) = 11.30, Z-score = 3.08, p = 0.001).

Subsequent post-hoc t-tests showed that patients exhibited left amygdala hyperactivation in response to neutral faces during the initial phase, compared to healthy controls (peak voxel xyz: -24 0–14, cluster size: 52, t(63) = 3.16, Z-score = 3.04, p = 0.001). During the second phase, no significant difference in left amygdala activation between patients and controls emerged.

#### Activation of the right amygdala in response to all faces as a function of group and phase

**Figure 1:**
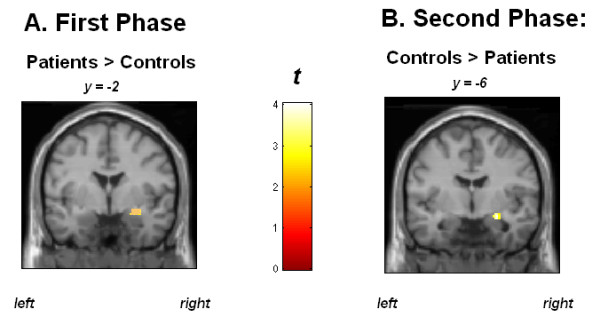
**Schizophrenia patients manifest increased right amygdala response to faces (versus no facial expression) compared to healthy controls during the first phase of the experiment (A) and healthy subjects show increased right amygdala activation in response to faces compared to patients during the second phase (B).** Blood oxygenation level-dependent responses are superimposed on MNI standard brain template. Coordinates in the reference frame of MNI space. Color bar: *t*-values.

To clarify the group*phase interaction effect, we compared the right amygdala response to all faces (angry, happy, and neutral) between groups for both phases of the experiment (see Figure 1). In the initial phase, patients manifested stronger amygdala activation to faces than did controls (peak voxel xyz: 30–2 -12, cluster size: 68, t(63) = 2.65, Z-score = 2.63, p = 0.004). In the second phase, healthy participants manifested stronger amygdala activation to faces than patients (peak voxel xyz: 26–6 -14, cluster size: 55, t(63) = 4.03, Z-score = 3.98, p = 0.00007).

## Discussion

The present study revealed new insights into abnormalities in the perception of facial emotion at an automatic processing level in schizophrenia patients. None of the participants included in our analyses had subjective awareness of emotional primes. Therefore, the emotional expression in prime faces was most likely processed automatically, without time for overt attention. As hypothesized, in response to angry, happy, and neutral faces versus no facial expression primes, we observed increased right amygdala activation in schizophrenia patients compared to healthy controls during the initial phase. For the left amygdala, we found a heightened response in patients only to neutral faces compared to controls. These results are consistent with those of Rauch et al. [16] and indicate a stronger automatic amygdala response to facial expression in schizophrenia. Initial hyperactivation of the amygdala in schizophrenia may indicate greater automatic encoding and an increased allocation of resources for processing facial expressions. It is well known that the amygdala modulates vigilance to enhance subsequent information processing throughout the brain [22], such that it represents an integral part of a cerebral network for unconscious emotional vigilance [23].

As hypothesized, patients showed an initial bilateral amygdala hyperresponsivity to masked neutral faces compared to healthy controls. Several previous studies have demonstrated that schizophrenia patients exhibit amygdala hyperactivation to neutral faces in paradigms using unmasked face stimuli [15,24]. The present findings suggest that this potential neural correlate of a generalized “fear of faces” [25] also emerges under automatic processing conditions. Therefore, previous results in studies using neutral faces as a baseline condition may be distorted [3,15]. Against this background, it is not surprising that Das et al. [14], who used neutral faces as a baseline, observed amygdala hypoactivation to masked fearful faces in schizophrenia patients compared to healthy controls.

Our hypothesis that amygdala activation in response to all facial primes would be reduced in the second compared to the first phase of the experiment was confirmed by the observed main effect of phase. These effects of time could be due to habituation upon the second presentation of the stimuli. Repetition suppression has previously been observed in the amygdala for subliminally presented facial emotions [26], and repetition-related reductions in amygdala responses to emotional stimuli have been demonstrated [19,20,27]. According to our data, the amygdala response to facial expressions rapidly habituates, even when faces are presented outside of subjects’ conscious awareness.

Our fMRI results corroborate the hypothesis that schizophrenia patients manifest a stronger decrease in the (right) amygdala response to facial stimuli over time than healthy individuals. During the first phase, patients showed stronger activation of the amygdala in response to faces than did controls, whereas in the second phase of the experiment, healthy individuals manifested stronger amygdala activation than patients. The decreases in amygdala activation observed in patients can be viewed in the context of their high levels of initial activation. The mechanisms underlying these effects of repetition and time may reflect an initial hypersensitivity to facial emotions and a subsequent down-regulation of activity. It appears that the processing of emotional faces is automatically suppressed over time in patients. This suppression could represent a compensatory mechanism for initial limbic hypersensitivity. The switch of patients from amygdala hyperactivation to hypoactivation (compared to healthy subjects) might reflect inhibitory processes. Blocking of amygdala responsivity might protect schizophrenia patients from overstimulation during social interactions.

Our findings on the automatic processing of emotional faces are consistent with observations from research on controlled emotion processing in schizophrenia. According to Holt et al. [15] and Salgado-Pineda et al. [9], schizophrenia patients exhibit amygdala activation that is similar or higher than in healthy individuals during the first stage of processing emotional information, and this activation subsequently diminishes more strongly than in healthy controls. Our data are in agreement with the assumption of Holt and Phillips [21] that emotional processing in schizophrenia could be characterized by an initial hyperresponsivity followed by hyporesponsivity of medial temporal lobe structures such as the amygdala.

To draw stronger conclusions, our finding of a switch from amygdala hyperactivation to hypoactivation in schizophrenia should be replicated. Further studies should examine the time-course of limbic activation during automatic as well as controlled processing of emotionally relevant stimuli in more detail. In addition, re-analyses of prior studies with a focus on time-course could provide valuable information. In particular, studies reporting amygdala under-recruitment during emotional face processing should be reconsidered [5,28], as their results might be explained at least in part by the occurrence of suppression processes over time.

According to our behavioral data, patients’ ratings were negatively biased by angry facial expressions. In the initial phase, patients’ evaluative ratings of neutral mask faces tended to be more negative when preceded by angry faces than when preceded by no facial expression primes, compared to healthy individuals. These findings corroborate previous results from affective priming studies, suggesting that schizophrenia patients exhibit a stronger negative evaluative bias in response to covert negative facial expressions than do healthy individuals [12,13]. The fact that affective priming due to masked angry faces decreased from the first to the second phase, independently of group, substantiates previous findings on the time-course of affective priming, with strong effects of masked emotional faces in initial presentations followed by substantial decreases in priming with repetition [18].

Some limitations of our study need to be acknowledged. First, our operationalization of time-course by dividing the experiment into two equivalent halves, with prime stimulus repetition between halves, was rather coarse. We analyzed ten events per condition and phase, but amygdala blocking might occur even earlier, possibly after only two or three presentations. More fine-grained experimental designs and time-sensitive methods such as EEG-informed fMRI might help uncover precisely when blocking processes begin. All patients in our study received atypical antipsychotics. Future research must clarify the effects of antipsychotic medication on the time-course of activation of the amygdala during emotional face processing. There is evidence from longitudinal fMRI research that treatment with atypical antipsychotics is associated with a reduction of the amygdala response during emotional processing in schizophrenia [29]. Against this background, antipsychotic medication in our sample could have counteracted the initial hyperresponsiveness effects observed in the patients in our study, which might therefore be even stronger in unmedicated patients. Furthermore, our sample size was too small to warrant subgroup analyses based on predominant negative or positive symptoms. A promising future direction may be to compare the neural underpinnings of automatic emotion processing in patient groups suffering primarily from anhedonia, affective flattening, or paranoid ideation. A plausible hypothesis is that the initial amygdala hyperactivation is most pronounced in patients suffering predominantly from positive symptoms such as persecutory delusions. Moreover, one could assume that the observed blocking of the amygdala response might be strongest in patients exhibiting severe affective negative symptoms.

## Conclusion

In the present study, we investigated amygdala responses to facial expression at an automatic processing level in schizophrenia. To examine the time-course of automatic emotion processing, our fMRI priming experiment was split into two halves, in which identical facial expressions were presented. As hypothesized, across priming conditions and groups, we observed a reduction of amygdala activation during the second compared to the first phase of the experiment. Schizophrenia patients are characterized by high initial amygdala responsivity to facial expression at an automatic processing level, which substantially diminishes over time. High initial activation of the amygdala to faces in schizophrenia could indicate a greater degree of automatic encoding and increased allocation of resources to processing these social stimuli. Amygdala deactivation over time might reflect an automatic mechanism by which schizophrenia patients suppress the processing of facial stimuli. This blocking mechanism could help patients avoid overstimulation during face-to-face contact. Finally, compared to healthy individuals, schizophrenia patients manifested initially enhanced bilateral amygdala responses to faces with neutral expression. Schizophrenia patients may perceive neutral facial expressions as ambiguous social cues, and the use of neutral faces as a baseline condition may therefore be inadequate for future neuroimaging research on the automatic processing of facial emotion in schizophrenia.

## Methods

### Participants

The original sample included 36 schizophrenia patients and 42 healthy controls, aged between 18 and 55 years. For all participants, exclusion criteria were a history of neurological disease, severe head trauma causing loss of consciousness, substance abuse during the last six months, and the usual magnetic resonance imaging contraindications. All subjects had normal or corrected-to-normal vision. Control subjects were thoroughly investigated by trained psychologists and were free of any lifetime history of psychiatric disorders, as diagnosed with the Structured Clinical Interview for DSM-IV, Axis I disorders [29]. Patients’ diagnoses were established by senior psychiatrists and confirmed by trained interviewers with the Structured Clinical Interview for DSM-IV [30]. During the patient interview, special attention was given to affective disorders to exclude a current depressive episode in study patients. In addition, all patients were administered a structured protocol of the Scale for the Assessment of Negative Symptoms (SANS) [31] and the Scale for the Assessment of Positive Symptoms (SAPS) [32]. For all participants, depressive symptoms were measured with the Beck Depression Inventory (BDI) [33]. Verbal intelligence was estimated by the Mehrfachwahl–Wortschatz–Intelligenztest (Multiple-Choice–Vocabulary–Intelligence Test; MWT-B) [34].

Five patients and six controls had to be excluded due to excessive movement during the functional scans or due to technical problems with the response boxes. One patient and one control subject had to be excluded because they reported (subjective) awareness of the primes, leaving a final sample of 30 patients and 35 controls. Table 2 lists sociodemographic and questionnaire data of the included participants and clinical data for patients. All 30 patients received second-generation antipsychotics, and three received additional typical antipsychotics. Thirteen patients were taking concomitant antidepressant medication, three were taking anticonvulsants, and one was taking lithium.

**Table 2:** Sociodemographic, handedness, intelligence, and affectivity data of patients (N = 30) and controls (N = 35), and clinical data for patients; mean ± SD (range); p: significance of two sample t-test (or chi-square test) comparing patients and controls

	**Patients**	**Controls**	**p**
Age	30.9 ± 7.6 (18–51)	29.6 ± 8.4 (19–49)	0.515
Education years	13.3 ± 2.4 (9–18)	14.6 ± 2.4 (9–18)	0.041*
Parental education years^1^	14.6 ± 2.8 (10–18)	14.9 ± 2.7 (11–18)	0.74
Sex (m/f)	17/13	23/12	0.61
Handedness (right/left)	29/1	33/2	1.000
Verbal intelligence^2^	108.1 ± 14.2 (88–136)	112.5 ± 11.9 (95–136)	0.17
BDI	12.5 ± 7.2 (0–28)	2.1 ± 2.8 (0–10)	<0.001*
STAI-T	47.4 ± 9.0 (25–71)	29.9 ± 6.5 (22–45)	<0.001*
SANS^3^ – flat affect	2.2 ± 1.2 (0–5)		
SANS – alogia	1.8 ± 1.0 (0–4)		
SANS – apathy	2.1 ± 0.6 (1–3)		
SANS – anhedonia	2.0 ± 1.0 (0–4)		
SANS – attention	1.9 ± 0.7 (0–3)		
SAPS – hallucinations	0.3 ± 0.5 (0–2)		
SAPS – delusions	1.2 ± 0.8 (0–3)		
SAPS – bizarre behavior	0.7 ± 0.8 (0–2)		
SAPS – formal thought disorders	1.8 ± 1.1 (0–4)		
Years of illness	6.7 ± 5.7 (0.5-19.0)		

^1^Years of education of the parent with the highest degree. Data missing for two patients.

^2^Assessed with the Mehrfachwahl-Wortschatz-Intelligenztest (MWT-B [33]).

^3^All SANS and SAPS scores represent global ratings of the symptom.

*p < .05 (two-tailed).

The study was conducted in accordance with the Declaration of Helsinki as revised in 1989 and was approved by the Ethics Committee of the University of Münster. After a complete description of the study, written informed consent was obtained. Patients received a financial compensation of 50 €, and controls received 40 €.

### Experimental design

**Figure 2:**
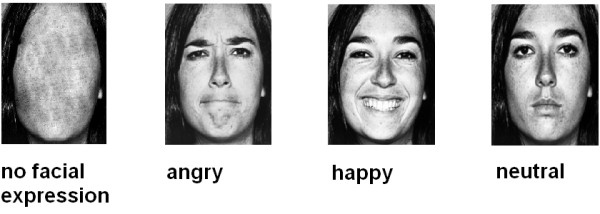
Examples of faces presented in the four prime conditions (happy, angry, neutral, and no facial expression).

**Figure 3:**
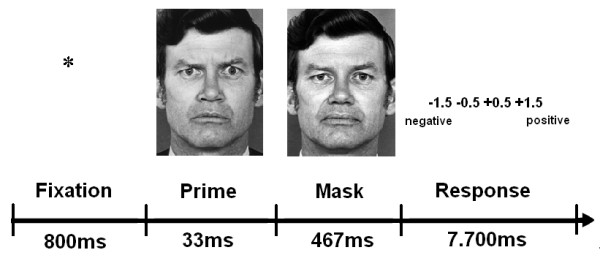
**Sequence of events within trials of the fMRI experiment.** Subjects had to evaluate the neutral mask face as expressing negative or positive feelings by pressing one of four buttons. In our example, a trial with an angry prime is shown.

Participants viewed gray-scale normalized face stimuli that were selected from a standardized picture set [35]. Ten pictures of neutral facial expressions (5 male and 5 female) were used as target stimuli. Prime stimuli were pictures of the same actors showing angry, happy, and neutral facial expressions. To avoid identity of prime and mask in the neutral face condition, neutral primes were vertically mirrored. As a baseline condition, we used no facial expression prime stimuli, consisting of neutral faces in which central facial features (i.e., eyes, nose, and mouth) had been replaced by a surface without contours (see Figure 2 for examples of the four prime conditions). Each trial lasted for 9 seconds. A fixation cross presented for 800 ms preceded a prime face shown for 33 ms, followed by the corresponding neutral face mask for 467 ms. A blank screen followed for 7.7 seconds (see Figure 3). Subjects were instructed to evaluate whether the neutral (mask) faces expressed rather negative or rather positive feelings, by pressing one of four buttons (− 1.5, − 0.5, + 0.5, and + 1.5). Judgments and reaction times were registered.

The paradigm consisted of two experimental phases, each with 40 trials: 10 with angry, 10 with happy, 10 with neutral, and 10 with no facial expression primes. In both phases, we used stimuli of the same ten actors, which were presented in a pseudo-randomized order. The transition from the initial phase (first half) to the second phase (second half) was not marked. The experiment lasted 12 min. Thus, our paradigm was a repetition priming paradigm with 40 stimuli presented in the first half of the experiment and the same 40 stimuli presented in the second half of the experiment. Stimuli were shown in a fixed pseudo-random order format. That means that randomization was implemented with the restriction of no repetition of an individual (taken from the Pictures of Facial Affect [35]) and no more than one repetition of a prime condition (angry, happy, neutral, or no facial expression) on consecutive trials.

To assess the success of masking, subjects were questioned after the experiment as to whether they had noticed anything out of the ordinary, and whether they had seen anything just before the neutral target faces. None of the participants included in the subsequent statistical analyses reported noticing emotional facial expressions prior to presentation of the neutral faces.

Behavioral affective priming scores were calculated by subtracting the mean evaluation score for the no facial expression prime condition from the mean evaluation scores for angry, happy, and neutral prime stimuli. Thus, negative scores indicate a negative evaluative bias and positive scores indicate a positive evaluative bias. Data were analyzed with a 2 (group)*2 (phase)*3 (condition) ANOVA using SPSS (IBM, Armonk, New York). Group (patients versus controls) was a between-subjects factor, and phase (initial versus second) and condition (angry versus happy versus neutral) were within-subjects factors. To overcome violations of sphericity in the data, the Greenhouse-Geisser statistic is reported for repeated-measures effects.

### fMRI methods

Images were projected to the rear end of the scanner (SharpXG-PC10XE with additional high frequency shielding; Osaka, Japan). T2* functional data were acquired in a 3-Tesla scanner (Gyroscan Intera 3.0 T, Philips Medical Systems, Best, NL) using a single shot echoplanar sequence, with parameters selected to minimize distortion in the regions of central interest while retaining adequate S/N and T2* sensitivity. Volumes consisting of 36 axial slices were acquired (matrix 64 * 63, resolution 3.6 * 3.6 * 3.5 mm; repetition time = 2.5 s, echo time = 35 ms, flip angle = 90°) 288 times in a slow event-related design.

Functional imaging data were motion-corrected using a set of six rigid body transformations determined for each image, spatially normalized to standard MNI space (Montreal Neurological Institute) with a voxel size of 2 * 2 * 2 mm and smoothed (Gaussian kernel, 8 mm FWHM) using Statistical Parametric Mapping (SPM5, Wellcome Department of Cognitive Neurology, London, UK). Statistical analysis was performed by modeling the different perceptual conditions (angry, happy, neutral, no facial expression) as variables within the context of the general linear model convolved with a standard hemodynamic response function. Fixed-effects analyses were performed at the individual level to generate individual contrast maps, and random effects analyses were performed at the group level. On a single subject level, contrast images were calculated for each of the two phases comparing each facial expression condition (angry, happy, neutral) with the no facial expression baseline condition, resulting in six contrast maps.

### Second-level fMRI analysis

Standard univariate group-level statistics were conducted using SPM5. For all analyses, the amygdala was chosen as the a priori region of interest (ROI). The amygdala was defined according to Tzourio-Mazoyer et al. [36], and the amygdala mask was created by means of the WFU PickAtlas [37]. To control for multiple statistical testing, we maintained a cluster-level false-positive detection rate at p < 0.05 using an uncorrected voxel threshold of p < 0.05 with a cluster extent (k Voxels) empirically determined by Monte Carlo Simulations (N = 1000 iterations) for the bilateral amygdala. This was performed by means of the AlphaSim procedure, which accounts for spatial correlations between BOLD signal changes in neighboring voxels implemented in the REST toolbox (http://restfmri.net/forum/index.php), yielding an empirically determined cluster threshold of k = 37 voxels.

Second-level fMRI data analyses were performed using SPM5. First, we computed a 2*2*3 full factorial ANOVA model with one between-subjects factor (group: patients versus controls) and two within-subjects factors (phase (first phase versus second phase) and condition (angry versus happy versus neutral face)) for the bilateral amygdala. Next, to clarify the significant group*phase*condition interaction found for the left amygdala, we computed separate 2 (group)*2 (phase) ANOVAs for each of the three facial expression conditions (i.e., angry, happy, and neutral). If required, t-tests for independent samples were subsequently applied to compare amygdala activation between groups for specific facial conditions and phases. Finally, to further analyze the significant group*phase interaction found in the initial ANOVA for the right amygdala, we compared activation of the right amygdala between groups in the initial and then in the second phase on all facial expression conditions by modeling the relevant between-group contrasts (all faces vs. no facial expression).

## Competing interests

All authors declare that there are no competing interests.

## Authors’ contributions

Conceived and designed the experiments: TS, HK, and CL. Performed the experiments: CL, MR, KW, and JB. Performed patient recruitment and established patient diagnoses: CL, BM, and RL. Analyzed the data: TS, CL, and UD. Contributed materials and analysis tools: BM, JB, PO, RL, AK, WH, VA, and HK. TS and CL drafted the manuscript, and all authors revised it critically for important intellectual content. All authors approved the final manuscript.
